# Growth Optimization and Secondary Metabolites Evaluation of *Anabaena variabilis* for Acetylcholinesterase Inhibition Activity

**DOI:** 10.3390/plants11060735

**Published:** 2022-03-10

**Authors:** Dina A. Refaay, Mohammed I. Abdel-Hamid, Amal A. Alyamani, Mamdouh Abdel Mougib, Dalia M. Ahmed, Amr Negm, Amr M. Mowafy, Amira A. Ibrahim, Rania M. Mahmoud

**Affiliations:** 1 Botany Department, Faculty of Science, Mansoura University, Mansoura 35516, Egypt; mhamid@mans.edu.eg (M.I.A.-H.); ammr79@mans.edu.eg (A.M.M.); 2 Department of Biotechnology, Faculty of Sciences, Taif University, P.O. Box 11099, Taif 21944, Saudi Arabia; a.yamani@tu.edu.sa; 3 Chemistry Department, Faculty of Science, Mansoura University, Mansoura 35516, Egypt; mmdhbdlmgb@hotmail.com (M.A.M.); amrbiochem@gmail.com (A.N.); 4 Department of Pharmaceutical Chemistry, Faculty of Pharmacy, Ain-Shams University, Cairo 11566, Egypt; daliamoged@pharma.asu.edu.eg; 5 Department of Chemistry, College of Science, King Faisal University, Al-Ahsa 31982, Saudi Arabia; 6 Department of Biological Sciences, Faculty of Science, New Mansoura University, New Mansoura City 35511, Egypt; 7 Plant Protection and Biomolecular Diagnosis Department, Arid Lands Cultivation Research Institute, City of Scientific Research and Technological Applications, New Borg El-Arab City 21934, Egypt; 8 Botany Department, Faculty of Science, Fayoum University, Fayoum 63514, Egypt; rmm00@fayoum.edu.eg

**Keywords:** acetylcholinesterase, *Anabaena variabilis*, Plackett–Burman, 5,7-dihydroxy-2-phenyl-4H-chromen-4-one, 4-phenyl-2-(pyridin-3-yl) quinazoline

## Abstract

Cyanobacteria comprise a good natural resource of a potential variety of neuro-chemicals, including acetylcholinesterase inhibitors essential for Alzheimer’s disease treatment. Accordingly, eight different cyanobacterial species were isolated, identified, and evaluated on their growth on different standard nutrient media. It was found that the modified *Navicula* medium supported the highest growth of the test cyanobacteria. The effects of methylene chloride/methanol crude extracts of the test cyanobacteria on acetylcholinesterase activity were examined and compared. *Anabaena variabilis* (KU696637.1) crude extract recorded the highest acetylcholinesterase inhibition (62 ± 1.3%). *Navicula* medium chemical components were optimized through a Plackett–Burman factorial design. The biomass of *Anabaena variabilis* increased significantly when grown on the optimized medium compared to that of control. The chemical analysis of the fractions derived from *Anabaena variabilis* showed the presence of two compounds in significant amounts: the flavonoid 5,7-dihydroxy-2-phenyl-4H-chrome-4-one and the alkaloid 4-phenyl-2-(pyridin-3-yl) quinazoline. Molecular docking studies revealed that both compounds interact with the allosteric binding site of acetylcholinesterase at the periphery with π-π stackings with Tyr341 and Trp286 with good, predicted partition coefficient. The compounds obtained from this study open the door for promising drug candidates to treat Alzheimer’s disease for their better pharmacodynamics and pharmacokinetic properties.

## 1. Introduction

Cyanobacteria produce an impressive array of natural substances, including polysaccharides, lipids, proteins, vitamins and sterols. Cyanobacteria are a feasible source of novel active drugs. Various cyanobacteria secondary metabolites were employed as antimicrobial, anti-inflammatory, antiviral, and anticancer agents [1,2]. As a source of neurochemicals, several studies have investigated the potential acetylcholinesterase (AChE) inhibition role of cyanobacteria extracts and compounds. For example, methanolic extracts from *Calothrix* sp., *Tolypothrix* sp., *Phormidium* sp., and *Geitlerinema splendidum* exhibited reversible inhibition of AChE [3]. Anatoxin-a(s) produced by several cyanobacteria species has shown potent AChE inhibition activity [4]. Furthermore, nostocarboline derived from *Nostoc* 78-12A was evaluated as an inhibitor for AChE [5].

Alzheimer’s disease (AD) represents one of the most widespread neurodegenerative disorders, and 10–15% of elderly people suffer from different forms of dementia [6]. AD is caused by a complex network of mechanisms and factors, all of which influence the progression of the disease [7].

Regarding the molecular mechanism of Alzheimer’s disease, two major hypotheses have been presented. The amyloid cascade and tau theory describes the process where beta-amyloid and tau proteins collected at synapses leads to a lack of nutrient supply and ultimately the death of neurons [8]. The cholinergic hypothesis, details the process where cholinergic neurons deteriorate, resulting in a lack of acetylcholine (ACh) and cholinergic loss [9]. In late stages of AD, acetylcholinesterase (AChE), the enzyme that terminates the function of the neurotransmitter acetylcholine, shows aberrant activity, leading to cholinergic system failure. AChE possesses an extremely fast turnover rate, which has been attributed to its unusually strong electric field that would attract cationic substrate and expel the anionic acetate product [10,11]. 

Although the actual cause of Alzheimer’s disease remains unknown, acetylcholinesterase inhibitors are the only medications approved to treat the symptoms of the disease [12]. Donepezil, [2-((1-Benzylpiperidin-4-yl) methyl)-5,6-dimethoxy-2,3-dihydro-1H-inden-1-one] for the treatment of mild and moderate AD, which interacts with residues of the anionic and peripheral sites of the enzyme rather than the exact binding site of acetylcholine, exhibits significant improvements when compared with the previous treatment of choice (tacrine) [13]. At the same time, prolonged use of such a medicine causes typical side effects such as nausea, diarrhoea, constipation, headache, dizziness, sleep problems, and stomach complications [14]. Thus, novel AChE inhibitors derived from natural sources such as cyanobacteria are required.

It is known that changes in culture conditions influence the secondary metabolism of cyanobacteria and can be used as a strategy to improve the production of compounds of interest which can be manipulated by statistical methods that involve changing one independent variable at a time while keeping others at a fixed level [15,16]. In this regard, Plackett–Burman design represents a well-established and widely used statistical method for optimizing the chemically definite medium and to screen which medium components provide maximum growth [17].

The current work aims to assess the inhibitory effect of crude extracts derived from different cyanobacterial species against AChE after optimizing biomass production of the promising isolate by Plackett–Burman factorial design. For this purpose, GC-MS, H-NMR, in vitro AChE inhibition assays, and molecular docking studies were employed.

## 2. Materials and Methods

Acetylcholinesterase (electric-eel AChE), acetylthiocholine iodide (ATCI) and 5, 5_dithio-bis-[2-nitrobenzoic acid] (DTNB) were purchased from Sigma. All other chemicals were of analytical grade. 

### 2.1. Cyanobacteria Isolates 

Eight different cyanobacterial species were isolated from the River Nile system at the Delta region. Three strains belonged to *Anabaena anomala* and the others to *Anabaena khannae*, *Anabaena varibilis* var. *ellipsospora*, *Anabaena variabilis*, *Anabaena fertilisma*, *Anabaena oryzae*, *Nostoc entophytum* and *Oscillatoria cortiana*. The isolates were identified according to the approved protocols [18,19]. Cyanobacteria were grown in axenic cultures of BG11 medium [20] at 25 ± 2 °C under continuous light of 45 μmol photons m^−2^ s^−1^.

### 2.2. Molecular Identification and Phylogenetic Analysis of Anabaena variabilis Based on 16S Ribosomal RNA Analysis

The genomic DNA of *A. variabilis* isolate (the most produced of secondary metabolites) was extracted to be the isolated fungal mat and was subjected to DNA extraction using DNeasy PowerSoil HTP 96 Kit (QIAGEN, Hilden, Germany). DNA concentration and purity was measured using a Nanodrop spectrophotometer (SPECTROstarNano, Germany). The PCR reaction mixture consisted of 1× buffer (Promega), 15 mM MgCl_2_, 0.2 mM dNTPs, 20 picomoles of 16S primer using the forward primer (5′ ACCTCCTTTCTAAGGAGCACC 3′) and the reverse primer (5′ GATGCTCGCAACCACTATCCA 3′) [21], 1u of Taq DNA polymerase (GoTaq, Promega), 40 ng DNA, and ultra-pure water to a final volume of 50 μL. PCR amplification was performed in a Perkin-Elmer/GeneAmp^®^ PCR System 9700 (PE Applied Biosystems) programmed to fulfill 35 cycles after an initial denaturation cycle for 5 min at 95 °C. Each cycle consisted of a denaturation step at 95 °C for 30 s, an annealing step at 51 °C for 30 s, and an elongation step at 72 °C for 30 s. In the final cycle, the primer extension segment was delayed to 7 min at 72 °C in the final cycle. The amplification parts were resolved by electrophoresis in a 1.5% agarose gel containing ethidium bromide (0.5 μL/mL) in 1X TBE buffer at 95 volts. The PCR products were subjected to a DNA purification kit (QIAGEN, Germany) and the purified DNA was subjected to DNA sequencing. The sequencing of the product PCR was carried out in an automatic sequencer ABI PRISM 3730XL Analyzer using Big Dye TM Terminator Cycle Sequencing Kits in an ABI 3730xl sequencer (Microgen Company). The relationships among sequences were analyzed using the BLAST program (http://www.ncbi.nlm.nih.gov/BLAST(7/7/2016 accessed on 6 January 2022). DNA nucleotide sequences were aligned using Align Sequences Nucleotide BLAST. The DNA nucleotide sequence for the *Anabaena variabilis* was banked in GenBank to obtain the accession numbers. MEGA X Software [22] was used to estimate the phylogenetic tree based on UPGMA (unweighted pair group method with arithmetic mean) statistical method (https://www.megasoftware.net/ accessed on 6 January 2022).

### 2.3. Effect of Different Nutrient Media on Growth of the Isolated Cyanobacteria

The growth of test cyanobacteria was assessed in three different standard nutrient media; BG11 medium [20], modified BG11 medium [23], and modified *Navicula* medium [24]. The initial inoculum of all tested cyanobacteria were equivalent to 0.005 g L^−1^ dry wt. 

### 2.4. Screening the Effect of Modified Navicula Nutrient Medium Components on Biomass Production of Anabaena variabilis by Plackett–Burman 

It is relevant to mention that *A. variabilis* maintains the highest AChE inhibition potential. Thus, Plackett–Burman experimental design was employed to test the effect of thirteen different factors (components of the modified *Navicula* nutrient medium) on the biomass production of *A. variabilis* including: Ca(NO_3_)_2_·4H_2_O, K_2_HPO_4_·3H_2_O, MgSO_4_·7H_2_O, Na_2_SiO_3_·9H_2_O, Na_2_CO_3_, H_3_BO_3_, MnCl_2_·4H_2_O, CuSO_4_·5H_2_O, HMoO_4_, ZnCl_2_, CoCl_2_·6H_2_O, FeCl_3_·6H_2_O, Na·EDTA·2H_2_O and one dummy factor to evaluate the standard error of the experiment. Each factor was tested at two levels: (−1) for a low level and (+1) for a high level Table 1. The level (−1) denotes a 50% chemical concentration of the variables, whereas (+1) denotes a 150 percent chemical concentration of the medium variables. The experiments were carried out in 250 mL Erlenmeyer flasks containing 100 mL medium. The flasks were inoculated with 0.01 g L^−1^ dry weight and incubated with shaking at 25 ± 2 °C for 15 days under continuous light of 45 μmol photons m^−2^ s^−1^. The main effect of each variable was determined with the following equation:

$$E_{\mathrm{xi}}=\frac{2[\sum \mathrm{Hxi}-\sum \mathrm{Lxi}]}{N}$$

The main effect with a positive sign indicates that the high concentration of this variable is nearer to optimum, and a negative sign indicates that the low concentration of this variable is nearer to the optimum.

### 2.5. Verification Experiment

To evaluate the accuracy of the applied Plackett–Burman design, the optimum levels of the screened variables for high growth of *Anabaena variabilis* were used to prepare a verification medium. The experiment was performed in plastic jars of 5 L volume capacity. The verified medium was inoculated with five days old culture with biomass equivalent to 0.01 g L^−1^ dry wt. and incubated static at the conditions described before. A control modified *Navicula* medium was also run in parallel. The biomass production was determined as freeze-dried weight (g L^−1^) after 15 days of the incubation period.

### 2.6. Biomass Harvesting

Known volumes of cyanobacteria cultures were collected after 15 days of growth, centrifuged at 2688× *g* for 10 min, and then the collected pellets were washed several times with glass-distilled water and frozen at −20 °C. Frozen pellets were then lyophilized and weighed [25].

### 2.7. Extraction with Methylene Chloride: Methanol (1:1) v/v

A sample of 3 g freeze-dried cyanobacteria was rolled in filter paper, gently pressed to render the biomass more fragile and porous, then placed in the reservoir of a Soxhlet extractor [26]. Methylene chloride: methanol in a ratio of 1: 1 (*v*/*v*) was used for extraction. The extraction process continued for 4 h and/or terminated when the solvent in the extraction reservoir became almost colorless. The extract was then evaporated under reduced pressure using a rotary evaporator (SENCO. Model R206D). The dry section was collected, weighed, and placed in clean dry glass vials and kept at −20 °C for further analyses.

### 2.8. Fractionation of Methylene Chloride: Methanol Crude Extract

The methylene chloride: methanol extract of *A. variabilis* 1:1 (*v*/*v*) was mixed with silica gel and placed as a band over the silica gel column. Elution of the column was performed using a series of eluents from hexane/ethyl acetate combinations of increase polarity. The effluents were combined into fractions according to their TLC patterns, and the final fractions were tested for their inhibition against AChE and the most promising fractions were further analyzed. 

### 2.9. In Vitro Acetylcholinesterase Activity Assay

The assay of AChE activity was slightly modified from the methods described previously [27]. The activity was measured by using a 96-well microplate with a 200 μL standard assay mixture containing 0.75 mM of 5, 5_-dithio-bis-[2-nitrobenzoic acid] (DTNB) (this concentration was recommended [28] to avoid its inhibitory effect on AChE activity), electric-eel AChE (0.0004 µg mL^−1^) in 50 mM Tris-HCl pH 8. The reaction was initiated by adding 1.5 mM of acetylthiocholine iodide (ATCI) as a substrate. The development of the yellow color of 5-thio-2-nitrobenzoate (TNB) (ϵ_405_ = 14.05 × 10^3^ M^−1^ cm^−1^) was monitored continuously at 405 nm in 25 °C. To test the inhibitory effect of different fractions, 5 μL of (10 µg mL^−1^) of each fraction was pre-incubated with the enzyme in the reaction mixture for one minute before initiating the reaction. The assay was also performed in the presence of 5 μL of the used solvent (methylene chloride/methanol in a ratio of 1:1 (*v*/*v*)) alone to monitor the effect of the solvent on the enzyme activity. Donepezil (10 µg mL^−1^) was used as positive control. The inhibition percent was calculated according to the following equation:

$$\mathrm{Inhibition}\;(\%)=\;\frac{A_{0}-A_{1}}{A_{0}}\times 100$$

where A_0_ is the enzyme’s activity when the solvent only was used (control), and A_1_ is the activity of the enzyme when the tested extract was used.

### 2.10. Analysis of the Chemical Constituents of Methylene Chloride: Methanol Extract

Detailed information regarding the crude methylene chloride: methanol extract of *A. variabilis* TLC fractionation and chemical characterization by ^1^H NMR and GC/MS analyses of the most promising fraction were mentioned in the Supplementary Materials. 

### 2.11. Molecular Docking Study of Certain Biochemical Constituents

Docking was initiated by retrieving the crystal structure of human recombinant AChE from the Protein Data Bank (PDB code: 4m0e). After removing all water molecules and repeated monomers, protein preparation was performed using Accelrys Discovery Studio Visualizer 2.5, which includes the application of CHARMm force field and then energy minimization using adopted basis NR algorithm. The enzyme’s binding pocket was identified as a sphere of radius 10 Å around dihydrotanshinone I (DHI) extending along the binding pocket. Docking of the prepared DHI using CDocker docking module was followed by alignment of the selected docked pose and the bioactive conformer to calculate RMSD. Docking of the ready test compounds using the same module and visual analysis of the 10 postures of each molecule was followed by re-scoring of the selected pose of each test molecule using other scoring functions.

### 2.12. Statistical Analysis

All experiments were performed in triplicates. Obtained data and results were expressed as mean ± standard deviation (±SD). All experiments were arranged in a completely randomized blocks design, and data were statistically analyzed by a one-way ANOVA test using Minitab 16 at *p* < 0.05 and 0.1.

## 3. Results

### 3.1. Growth of Different Cyanobacteria

**Figure 1:**
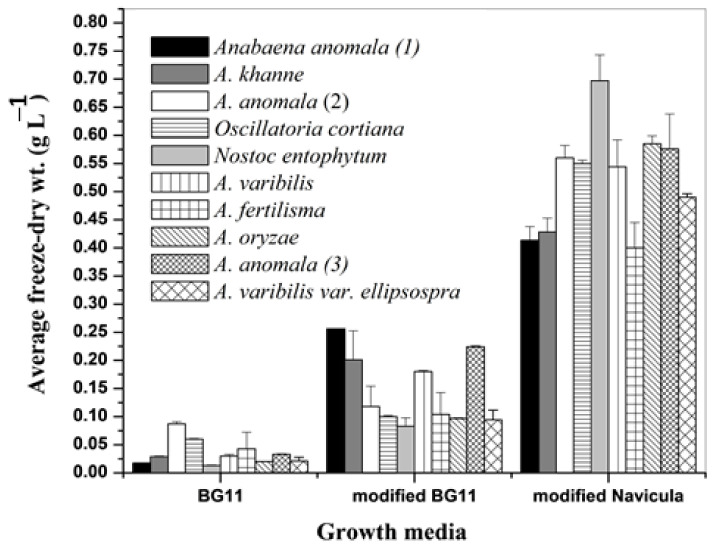
Average freeze-dry weight (mean of three replicates ± SD) of different species of cyanobacteria grown on different nutrient media.

The effect of different nutrient media on dry weight biomass production (g L^−1^) of other test cyanobacteria is illustrated in Figure 1. The modified *Navicula* medium supported relatively the highest growth of the tested cyanobacteria. The growth of different cyanobacteria fluctuated between 0.013 ± 0.001 and 0.087 ± 0.004 g L^−1^ in BG11, 0.083 ± 0.015 and 0.265 ± 0.013 g L^−1^ in modified BG11, 0.4 ± 0.045 and 0.697 ± 0.046 g L^−1^ in the modified *Navicula* media. Accordingly, the modified *Navicula* medium was chosen as best for growth of cyanobacteria for further research steps in this experiment. 

### 3.2. Inhibition of Acetylcholinesterase Activity by Tested Cyanobacteria Crude Extracts

The effects of crude extracts of different tested cyanobacteria are listed in the Table 2. Only three extracts obtained from *A. anomala* (1), *A. oryzae* and *A. variabilis* exhibited inhibitory effects upon AChE by 25 ± 0.87%, 49 ± 1.5% and 62 ± 1.3%, respectively. Based on these results, *A. variabilis* extract maintained the highest percent of inhibition, and thus became the focus of intention in the following steps.

### 3.3. Screening the Effect of Modified Navicula Nutrient Medium Component on Biomass Production of A. variabilis by Plackett–Burman Design

The results listed in Table S1 show that biomass obtained from different runs fluctuated between 0.878 g L^−1^ (run ≠ 11) and 4.028 g L^−1^ (run ≠ 13). Compared to control, all the experimental runs maintained either a non-significant or significant (*p* < 0.1) increase or decrease in dry weight. 

Some constituents of the control *Navicula* media reported maintaining positive and others negative effects on the growth of *A. variabilis*. The results of Plackett–Burman design experiment revealed that the impact of the variables; MgSO_4_·7H_2_O, Na_2_SiO_3_·9H_2_O, Na_2_CO_3_, H_3_BO_3_, MnCl_2_·4H_2_O, CuSO_4_·5H_2_O, HMoO_4_, CoCl_2_·6H_2_O and FeCl_3_·6H_2_O bear a positive effect on the growth of *Anabaena variabilis* and indicated that the maximum biomass production of *Anabaena variabilis* required 1.5-fold concentrations of these components compared to that of control medium (run 21). In contrast, the main adverse effect of the variables; Na_2_EDTA·2H_2_O, ZnCl_2_, Ca(NO_3_)_2_·4H_2_O and K_2_HPO_4_·3H_2_O indicated that *A. variabilis* required half concentrations of these constituents compared to the control medium for maintaining the highest biomass production. The statistical analyses of the main effect of thirteen different constituents of the nutrient medium are given in Table 3. 

### 3.4. Verification Experiments

Based on these results, it can be concluded that, the best medium composition (g L^−1^) of the modified *Navicula* medium providing the highest biomass production for *Anabaena variabilis* is as follows: Ca(NO_3_)_2_·4H_2_O (0.05 g L^−1^), KHPO_4_·3H_2_O (0.07), MgSO_4_·7H_2_O (0.0375), Na_2_SiO_3_·9H_2_O (0.15), Na_2_CO_3_ (0.03), H_3_BO_3_ (0.0042), MnCl_2_·4H_2_O (0.00135), CuSO_4_·5H_2_O (0.0012), HMoO_4_ (0.00135), ZnCl_2_ (0.00006), CoCl_2_·6H_2_O (0.00006), FeCl_3_·6H_2_O (0.075), Na·EDTA·2H_2_O (0.015). This medium can be designated as the verified or verification growth medium.

**Figure 2:**
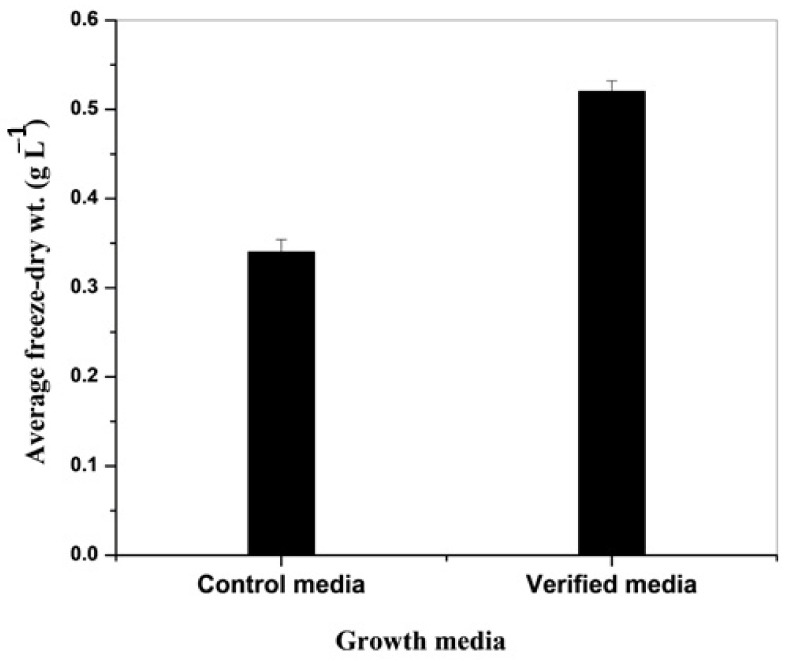
Average freeze-dry weight of *Anabaena variabilis* grown in control and verified (optimized) *Navicula* medium.

As seen from Figure 2, the biomass *Anabaena variabilis* following 15 days of growth increased significantly (*p* ≤ 0.05) when grown in the verification medium (0.52 ± 0.012 g L^−1^) compared to that of control *Navicula* medium (0.34 ± 0.014 g L^−1^).

### 3.5. Phylogenetic Analysis and Placement of Anabaena variabilis

**Figure 3:**
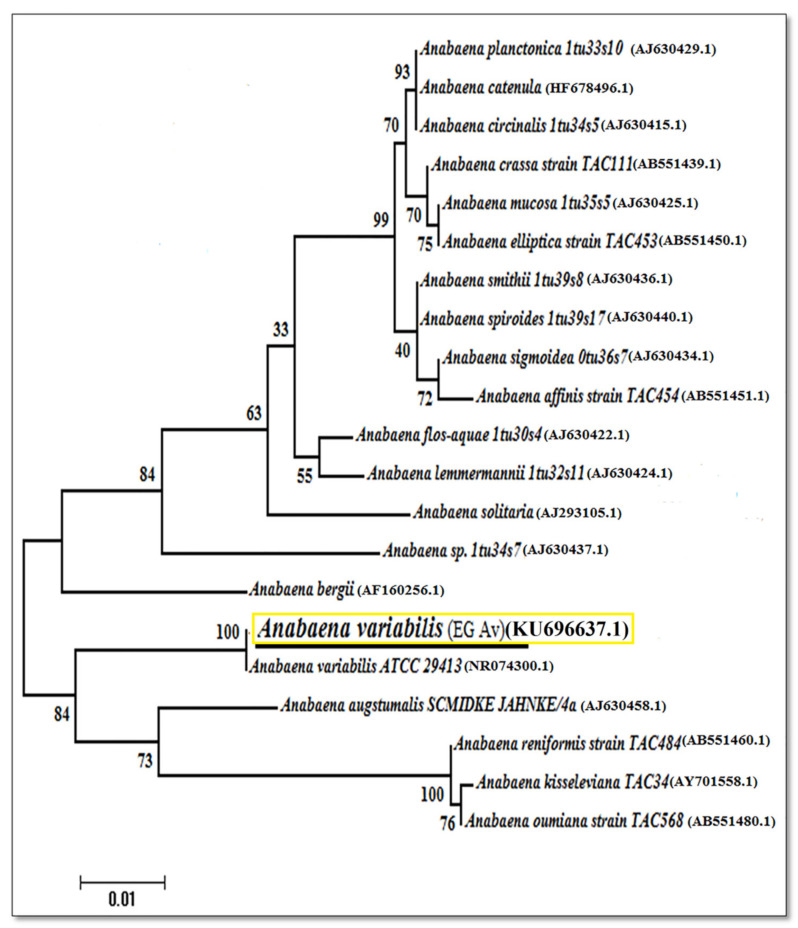
The phylogenetic tree was constructed via the bootstrap test of maximum-likelihood (ML) algorithm based on the 16S rRNA gene sequence of *A. variabilis* (highlighted in yellow).

*Anabaena variabilis* isolate was identified molecularly using the 16s mRNA region with accession number KU696637.1. The isolate displayed 98–100% similarity with the formerly nearby relative isolates in Genbank. Phylogeny cluster tree topologies of the sequence was demonstrated in (Figure 3). The Egyptian isolate *Anabaena variabilis* in this study falls in the same clade with *Anabaena variabilis* strain ATCC 29413 in USA with accession No. (NR_074300.1) with highest support values (100%) of bootstrap percentages (BP). 

### 3.6. The AChE Inhibitory Activity of Ten TLC Fractions

The (methylene chloride: methanol) extracts obtained from *A. variabilis* were separated and the effluents were combined into fractions according to their TLC patterns, and finally, 10 fractions were obtained. The AChE inhibition of 10 TLC fractions is listed in Table 4. Only four fractions exhibited inhibitory effects on AChE. F7 maintained the highest percent of inhibition (73.6%) followed by F8 (50%), F10 (45.2%) and F6 (32.8%). Based on these results, F7 maintained relatively the highest inhibitory effects; therefore, representative samples were sent for GC/MS analysis for structural elucidation of major biochemical constituents.

### 3.7. GC/MS Analyses of the Fraction F_7_

**Figure 4:**
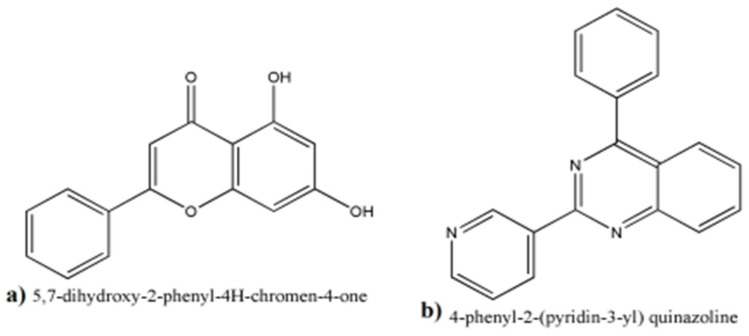
The chemical nature of some of the identified compounds in F7 (**a**) 5,7-dihydroxy-2-phenyl-4H-chrome-4-one and (**b**) 4-phenyl-2-(pyridin-3-yl) quinazoline compounds.

The GC chromatogram for F7 exhibited 54 peaks (Figure S1) corresponding to 54 compounds included (Table S2). The fraction contained compounds of an aromatic nature. The flavonoid 5,7-dihydroxy-2-phenyl-4H-chrome-4-one and the alkaloid 4-phenyl-2-(pyridin-3-yl) quinazoline were measured with considerable amounts 1.38% and 3.45%, respectively (Figure 4). 

### 3.8. Alignments and Docking Poses of DHI, Donepezil, 5,7-dihydroxy-2-phenyl-4H-chrome-4-one and 4-phenyl-2-(pyridin-3-yl) Quinazoline

**Figure 5:**
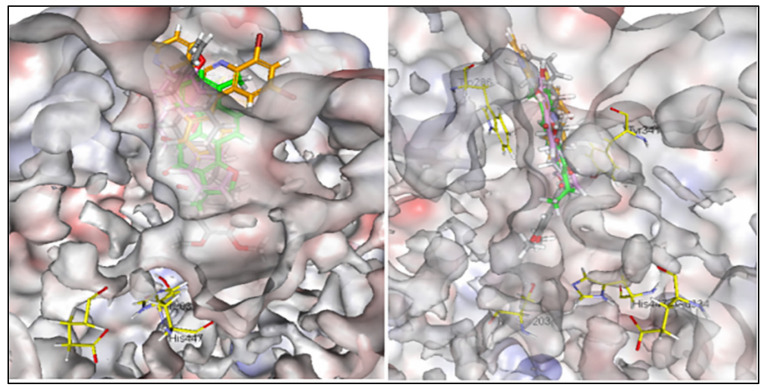
Alignments of DHI (green), donepezil (grey), 5,7-dihydroxy-2-phenyl-4H-chromen-4-one (pink) and 4-phenyl-2-(pyridin-3 yl)quinazoline (orange) in the active site of AChE (Left: face view, Right: side view).

Donepezil extends along the entire length of the enzyme active-site pocket (AChE) with other planer shaped inhibitors including the standard inhibitor dihydrotanshinone I (DHI). Two biochemical constituents of methylene chloride:methanol extract of *A. variabilis* (KU696637.1), namely 5,7-dihydroxy-2-phenyl-4H-chrome-4-one and 4-phenyl-2-(pyridin-3-yl) quinazoline were able to interact with the allosteric binding site of AChE at the periphery. The results are illustrated in Figure 5. The compounds 5,7-dihydroxy-2-phenyl-4H-chrome-4-one and 4-phenyl-2-(pyridin-3-yl)quinazoline provided 10 possible docked poses. The ideal pose of each molecule was selected according to the similarity of its binding mode in the binding site to that of DHI. 

**Figure 6:**
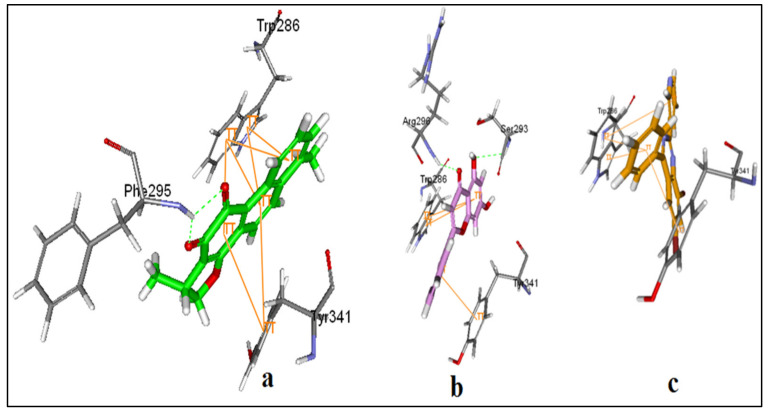
The 3D binding mode of DHI (**a**), 5,7-dihydroxy-2-phenyl-4H-chrome-4-one (**b**) and 4-phenyl-2-(pyridin-3-yl)quinazoline (**c**) showing π-π interactions as orange lines and hydrogen bonds as green dotted lines with the interacting amino acids (the rest of the binding site amino acids are hidden for simplification).

DHI interacts at the peripheral site with many π-π stackings with Tyr 341 and Trp 286. Moreover, the two carbonyl oxygens showed two hydrogen bonds with Phe 295 (Figure 6a). 5,7-dihydroxy-2-phenyl-4H-chrome-4-one shows similar π-π stackings with Tyr 341 and Trp 286. Additionally, a hydrogen bond is present between Arg 296 and carbonyl oxygen of length 2.2 Å. Moreover, two hydrogen bonds between 5-OH and Ser 293 of length 2.1 and 2.5 Å represent extra binding (Figure 6b). Although 4-phenyl-2-(pyridin-3-yl)quinazoline could not fit more profoundly in the peripheral site, it maintained π-π stackings with Tyr 341 and Trp 286 and could still block the entrance of the pocket (Figure 6c).

### 3.9. Binding Energies of the Selected Poses of DHI, Donepezil, 5,7-dihydroxy-2-phenyl-4H-chrome-4-one, and 4-phenyl-2-(pyridin-3-yl) Quinazoline

The scoring functions of the selected poses exhibited a higher binding energy value for 5,7-dihydroxy-2-phenyl-4H-chrome-4-one, designated in Table 5 as A compared to 4-phenyl-2-(pyridin-3-yl)quinazoline, designated in Table 5 as B. The scoring functions LigScore, PLP, and Jain estimate the affinity of the ligand to the receptor. These indicate more or less similar binding affinity of the biochemical constituents 5,7-dihydroxy-2-phenyl-4H-chrome-4-one, 4-phenyl-2-(pyridin-3-yl)quinazoline and both the standard ligand DHI and the drug donepezil (Table 5).

### 3.10. Pharmacokinetic Profiles of 5,7-dihydroxy-2-phenyl-4H-chrome-4-one and 4-phenyl-2-(pyridin-3-yl) Quinazoline

Pharmacokinetics of the test compounds are listed in Table 6. The logarithm of the partition coefficient (P) (A log P 98) of 5,7-dihydroxy-2-phenyl-4H-chrome-4-one (A) is better than that of 4-phenyl-2-(pyridin-3-yl)quinazoline (B). The polar surface area (PSA) was good for both 5,7-dihydroxy-2-phenyl-4H-chrome-4-one (A) and 4-phenyl-2-(pyridin-3-yl)quinazoline (B). Human intestinal absorption (HIA) of both compounds was predicted to be good while blood–brain barrier (BBB) penetration prediction was medium for 5,7-dihydroxy-2-phenyl-4H-chrome-4-one (A) and very good for 4-phenyl-2-(pyridin-3-yl)quinazoline (B). 5,7-dihydroxy-2-phenyl-4H-chrome-4-one (1) showed good aqueous solubility while 4-phenyl-2-(pyridin-3-yl)quinazoline (2) exhibited poor aqueous solubility. The results indicate that both compounds displayed potential hepatotoxicity. Moreover, both compounds are inhibitors of CYP2D6.

## 4. Discussion

Acetylcholinesterase inhibitors represent critical medications regarding Alzheimer’s disease treatment. Despite various commercial products, novel natural chemicals are still desperately needed. In this regard, cyanobacteria produce different secondary metabolites with potent AChE activities such as anatoxin-a(s) and nostocarboline [4,5]. The experimental results (Figure 1) indicated that the modified *Navicula* nutrient medium supported the highest growth of all tested cyanobacteria. It has been well documented [29,30] that the culture medium affects the cyanobacteria growth and the composition and yield of a specific metabolite. The marked increase in dry weight of all tested cyanobacteria grown in the modified *Navicula* medium could be primarily attributed to the composition of this medium, therefore, justified the selection of this medium for further growth experiments.

It has become evident that the extensive use of synthetic acetylcholinesterase (AChE) inhibitors such as donepezil, galanthamine, and rivastigmine for the conventional treatment of AD can result in a variety of side effects. Such side effects include liver damage, vomiting, nausea, and an increase in the frequency of bowel movements [31,32]. Therefore, exploring new drugs of a natural origin for the treatment of AD without side effects is urgently needed. 

The effect of methylene chloride/methanol extracts on AChE activity was either stimulatory or inhibitory (Table 2). The inhibition of AChE activity by three crude extracts may lead us to drive an anti-ACHE component(s) from *A. anomala* (1), *A. oryzae* and *A. variabilis*. In this regard, acetylcholinesterase inhibitors have been, and remain, the conventional approach to the symptomatic treatment of AD. It was reported that the treatment of early and moderate Alzheimer’s disease has largely involved the replacement of neurotransmitters that are known to be lacking, mostly based on AChE inhibition [33,34]. 

Our findings confirmed the observations of [35], who found potent AChE inhibition potential of the methanolic extracts of four *Nostoc* strains, *Nostoc* sp. str. Lukešová 27/97, *Nostoc ellipsosporum* Rabenh. str. Lukešová 51/91, *Nostoc ellipsosporum* str. Lukešová 52/91 and *Nostoc linckia f. muscorum* (Ag.) Elena. str. Gromov, 1988, CALU-980, in addition to [36] who noted reversible inhibition of AChE with the methanolic extracts of *Calothrix* sp. CBT 3320, *Tolypothrix* sp. CBT 3321, *Phormidium* cf. *amoenum* CCIBt 3412, *Phormidium* sp. CCIBt 3265, and *Geitlerinema splendidum* CCIB.3223. 

The experimental results indicated that *A. variabilis* (KU696637.1) maintained both the highest growth production on the modified *Navicula* nutrient medium (Figure 1) and AChE inhibition activity (Table 2). This cyanobacterium was the focus of further research to reveal its potential as a renewable bio-resource of new neurochemicals with possible AD management. 

The media type affected the growth of microalgae and the composition and, most significantly, the mass ratio between different components [36]. In this concern, a Plackett–Burman approach was designed to screen the most appropriate medium composition, giving the living cells the highest production, including microalgae [37]. 

In this study, Plackett–Burman design was employed to evaluate the significance of each medium component towards biomass production of *A. variabilis*. The experimental results (Figure 2) indicated that the maximum biomass production of *A. variabilis* on the optimized medium required 1.5-fold of MgSO_4_·7H_2_O, Na_2_SiO_3_·9H_2_O, Na_2_CO_3_, H_3_BO_3_, MnCl_2_·4H_2_O, CuSO_4_·5H_2_O, HMoO_4_, CoCl_2_·6H_2_O, FeCl_3_·6H_2_O and half the concentration of Na_2_EDTA·2H_2_O, ZnCl_2_, Ca(NO_3_)_2_·4H_2_O and K_2_HPO_4_·3H_2_O. In this regard, P, N, and Fe are the essential liming nutrients for cyanobacterial growth [38]. N_2_-fixing cyanobacteria may have different demands for P, Fe, and N than non-N_2_-fixing bacteria; limited quantities of inorganic N usually enhance N2-fixation (e.g., NH_3_ and NO_3_^−^), whereas high concentrations of inorganic N hinder N_2_-fixation [39]. Furthermore, several cyanobacteria at low phosphorus levels can store phosphates as polyphosphates in polyphosphate bodies (volutine granules) allowing them to divide twice or four times, resulting in a 4–32-fold increase in biomass [40]. On the other hand, cyanobacteria require significantly more Fe than eukaryotic algae. It has been reported [41,42] that, N_2_-fixing cyanobacteria bear a specific requirement for Fe nitrogenase, and Fe additions have been observed to boost growth rates, photosynthesis, and N fixation. The overall increase in dry weight biomass of *A. variabilis* on the optimized medium may be attributed to the products of additive, antagonistic and synergetic interactions among all the medium constituents. 

Since the composition of nutrient media affects biomass production and the composition and yield of specific metabolites [43,44], it seems essential to analyze the biochemical composition of *Anabaena variabilis* biomass grown on verified *Navicula* medium (optimized). Accordingly, the column chromatographic technique was applied to separate the mixture of compounds of *A. variabilis* crude extract. According to [45], column chromatography is one of the most valuable procedures for purifying chemicals that require polarity differences to be separated. To separate chemical compounds with more polar functional groups that potentially bear an inhibitory effect against AChE, a silica gel was used as an adsorbent (stationary phase). Different volumetric proportions (*v*/*v*) of hexane: ethyl acetate with increasing polarity was used as a mobile phase to elute the less polar to highly polar biochemical constituents [46].

Fractions of *Anabaena variabilis* crude extract grown on optimized *Navicula* medium were analyzed by ^1^H-NMR for determining the primary chemical composition of different fractions and at the same time to select fractions with possible bioactive functions for further GC/MS analysis. Fatty acids and/or fatty materials in almost all fractions except fractions 7 and 8 showed aromatic compounds (Table 4, Figures S2 and S3). As expected, when the assay of AChE was performed using all fractions, the marked inhibition was observed in fraction 7. This observation indicated that the targeted inhibitor(s) might be of an aromatic nature. Mukherjee et al. [32] stated that the major classes of compound with anticholinesterase activity include alkaloids, flavonoids and terpenoids. Accordingly, it seems relevant to match the biochemical constituents of F7 that was identified by GC/MS analysis with those reported to maintain AChE inhibitory effects in the literature.

Among the identified compounds of F7 (Table S2), The flavonoid 5,7-dihydroxy-2-phenyl-4H-chrome-4-one and the alkaloid 4-phenyl-2-(pyridin-3-yl) quinazoline (Figure 4) were closely similar to luteolin that was isolated from the leaves of *Morus alba* for its ability to act as an AChE inhibitor [47] and huperzine A, a quinazolidine alkaloid from *Huperzia serrata* that was identified for its anti-AChE effect [48], respectively. To the best of our knowledge, this study might be the first to identify these compounds from *A. variabilis*. The observed anti-AChE in fraction 7 containing these compounds beside the recent reports describing the anti-AChE of these compounds analogues together pushed us to confirm their inhibitory effect via molecular docking; including two well-known AChE inhibitory molecules namely, 2-((1-Benzylpiperidin-4-yl) methyl)-5,6-dimethoxy-2,3-dihydro-1H-inden-1-one (donepezil, FDA approved drug for AD treatment) and dihydrotanshinone I (DHI) were employed.

Due to the elongated structure of donepezil, it extends along the entire length of the enzyme active-site pocket, while dihydrotanshinone I (DHI), 5,7-dihydroxy-2-phenyl-4H-chrome-4-one, and 4-phenyl-2-(pyridin-3-yl)quinazoline (Figure 5) were able to interact with the allosteric binding site at the periphery. Accordingly, [49] stated that the binding site of AChE contains mainly two sites. The catalytic site, which contains the catalytic triad (His 447, Glu 334, and Ser 203) and is located at the base of the pocket and the peripheral site which extends beyond Tyr 337 and is located at the top of the pocket and provides a binding site for allosteric modulators inhibitors. Scoring function (Table 5) showed higher binding energy value for 5,7-dihydroxy-2-phenyl-4H-chrome-4-one as compared to 4-phenyl-2-(pyridin-3-yl)quinazoline. Cheung et al. [49] stated that one of the most important principles for designing or obtaining potential new ligands is to predict the binding affinity of a certain ligand to its target and use the predicted affinity as a criterion for selection.

Cheng et al. [50] and Podlogar et al. [51] stated that in the early stages of drug discovery from promising compounds, the early estimation of several ADMET properties represent essential issues before using this compound in clinical settings. In this way, the pharmacokinetic profiles of 5,7-dihydroxy-2-phenyl-4H-chrome-4-one and 4-phenyl-2-(14yridine-3-yl)quinazoline (Table 6) were very important to predict the suitability of both compounds in drug design for AD treatment. Authors of [52] stated that for optimum cell permeability drug should bear a polar surface area (PSA) <140 Å and partition coefficients (A log P 9) <5; thus 5,7-dihydroxy-2-phenyl-4H-chrome-4-one and 4-phenyl-2-(pyridin-3-yl)quinazoline were predicted to present with good partition coefficients. 

According to [53], the distribution or partition coefficient of medicine exerts a significant impact on how readily it may reach; its intended target in the body, how powerful of an effect it will bear once it reaches its target, and how long it will remain in an active form in the body. In this context, the poor aqueous solubility of 4-phenyl-2-(pyridin-3-yl)quinazoline (F_7_, Table 6) means that this hydrophobic compound will mainly be distributed to hydrophobic areas such as the lipid bilayers of cells while the better aqueous solubility of 5,7-dihydroxy-2-phenyl-4H-chrome-4-one estimates that this hydrophilic compound will primarily be found in aqueous regions such as blood serum [54,55].

It has been well documented [56,57] that for drugs targeted at the central nervous system (CNS), the BBB penetration is necessary. In contrast, BBB penetration may lead to undesirable adverse effects for drugs acting in peripheral tissues. Accordingly, the high BBB permeability of 4-phenyl-2-(pyridin-3-yl)quinazoline (F_7_, Table 6) confirms the suitability of this compound to inhibit AChE in the brain of AD patients.

Both 5,7-dihydroxy-2-phenyl-4H-chrome-4-one and 4-phenyl-2-(pyridin-3-yl)quinazoline (F_7_, Table 6) bind effectively with plasma proteins (PPB). It has been reported [58,59] that the degree of binding to plasma proteins (PPB) has a significant impact on the drug’s pharmacokinetic and pharmacodynamic properties and efficiency. Drugs that are bound to plasma proteins are not actively distributed to the site of action to interact with the target tissues; only the free drug interacts with receptors, producing their therapeutic effect. Moreover, the bound drug is retained in the blood, while the unbound (free) fraction may be metabolized or excreted.

## 5. Conclusions

Finally, it can be concluded that both 5,7-dihydroxy-2-phenyl-4H-chrome-4-one and 4-phenyl-2-(pyridin-3-yl) quinazoline might represent promising drug candidates for the treatment of AD, due to their better pharmacodynamics and pharmacokinetic properties. The deep kinetic analyses and in vivo experiments on animal models following isolation and purification of both compounds remain in our future considerations in accepting these compounds as drugs for Alzheimer’s diseases.

## Figures and Tables

**Table 1:** Variables investigated of the modified *Navicula* medium via Plackett–Burman design for *Anabaena variabilis*.

No	Factors	Variables	Zero Level (0)	Low Level (−)	High Level (+)
1	X1	Ca(NO_3_)_2_·4H_2_O	0.1	0.05	0.15
2	X2	K_2_HPO_4_·3H_2_O	0.14	0.07	0.21
3	X3	MgSO_4_·7H_2_O	0.025	0.0125	0.0375
4	X4	Na_2_SiO_3_·9H_2_O	0.1	0.05	0.15
5	X5	Na_2_CO_3_	0.02	0.01	0.03
6	X6	H_3_BO_3_	0.0028	0.0014	0.0042
7	X7	MnCl_2_·4H_2_O	0.0009	0.00045	0.00135
8	X8	CuSO_4_·5H_2_O	0.0008	0.0004	0.0012
9	X9	HMoO_4_	0.0009	0.00045	0.00135
10	X10	ZnCl_2_	0.00013	0.00006	0.00019
11	X11	CoCl_2_·6H_2_O	0.00004	0.00002	0.00006
12	X12	FeCl_3_·6H_2_O	0.005	0.0025	0.0075
13	X13	Na·EDTA·2H_2_O	0.03	0.015	0.045
14	X14	Dummy	-		

**Table 2:** AChE inhibition % in the presence of tested cyanobacteria crude extracts.

Cyanobacteria Isolates	% Inhibition	Effect
*Anabaena anomala* (1)	25 ± 0.87	Inhibitory
*Anabaena khanne*	-- *	Stimulatory
*Anabaena anomala* (2)	-- *	Stimulatory
*Oscillatoria cortiana*	-- *	Stimulatory
*Nostoc entophytum*	-- *	Stimulatory
*Anabaena variabilis*	62 ± 1.3	Inhibitory
*Anabaena fertilisma*	-- *	Stimulatory
*Anabaen oryzae*	49 ± 1.5	Inhibitory
*Anabaena anomala* (3)	-- *	Stimulatory
*Anabaena varibilis* var. *ellipsospora*	-- *	Stimulatory
Donepezil	100 ± 0.0	

* These crude extracts increase the activity of AChE (stimulatory).

**Table 3:** Statistical analysis of the results of Plackett–Burman experiment of each variable.

Variable	Effect	Coefficient	T-Value	*p*-Value	Significance
X1 (Ca(NO_3_)_2_·4H_2_O)	−0.4305	−0.215	−1.45	0.28	NS
X2 (K_2_HPO_4_·3H_2_O)	−0.0251	−0.0126	−0.07	0.94	NS
X3 (MgSO_4_·7H_2_O)	0.126	0.063	0.35	0.742	NS
X4 (Na_2_SiO_3_·9H_2_O)	0.155	0.077	0.43	0.68	NS
X5 (Na_2_CO_3_)	0.236	0.118	0.65	0.54	NS
X6 (H_3_BO_3_)	0.122	0.061	0.34	0.74	NS
X7 (MnCl_2_·4H_2_O)	0.472	0.236	1.59	0.135	NS
X8 (CuSO_4_·5H_2_O)	0.831	0.415	2.8	0.014	S
X9 (HMoO_4_)	0.337	0.168	0.93	0.39	NS
X10 (ZnCl_2_)	−0.568	−0.284	−1.91	0.076	S
X11 (CoCl_2_·6H_2_O)	0.2449	0.122	0.68	0.52	NS
X12 (FeCl_3_·6H_2_O)	0.7227	0.361	2.43	0.029	S
X13 (Na·EDTA·2H_2_O)	−0.383	−0.194	−1.06	0.33	NS

NS = non-significant at *p* < 0.1 and S = significant at *p* < 0.1.

**Table 4:** The chemical constituents of different fractions of *A*. *variabilis* extract and their corresponding effect on AChE activity.

Fractions	Components	% Inhibition
F1	Saturated fatty acids	-
F2	Saturated fatty acids	-
F3	Saturated fatty acids	-
F4	Saturated fatty acids	-
F5	Saturated fatty acids	-
F6	Saturated fatty acids	32.8
F7	Aromatic compounds, saturated and unsaturated fatty acids	73.6
F8	Aromatic compounds, saturated and unsaturated fatty acids	50
F9	Fatty materials	8
F10	Fatty materials	45.2
Donepezeil	-	100 ± 0.0

**Table 5:** Binding energies of the selected poses of DHI and test molecules.

Compound	CDOCKER Interaction Energy (Kcal mole^−1^)	CDOCKER Energy (Kcal mole^−1^)	Scoring Function				
			LigScore1	LigScore2	PLP1	PLP2	Jain
Donepezil	45.18	7.24	2.68	5.52	86.14	84.95	3.81
DHI	38.92	1.30	3.28	5.54	99.96	91.50	4.27
A	35.28	30.23	4.17	5.57	79.80	85.20	2.52
B	32.65	5.64	1.76	5.20	76.97	68.99	0.37

**Table 6:** ADMET prediction descriptors calculated by Discovery Studio 2.5.

ADMET Tests	ADMET Level	Donepezil	A	B
A log P 98	<5 (good)	4.12	2.652	5.301
PSA	<140 Å (good)	38.77	67.861	33.783
Absorption	0 (good)		0	0
BBB	0.9 (very good) 2 (medium)	0.9953	0.408	0.95
Solubility	1 (poor) 3 (good)		−3.259	−6.895
Hepatotoxicity	1 (toxic)		0.933	0.933
CYP2D6	1 (inhibitor)	0.8684	0.772	0.801
PPB level	2 (binding ≥ 95%)	96%	2	2

## Data Availability

Relevant data applicable to this research are within the paper.

## Supplementary Materials

The following supporting information can be downloaded at: https://www.mdpi.com/article/10.3390/plants11060735/s1, Figure S1: GC chromatogram of fraction F7; Figure S2: 1H-NMR of methylene chloride: methanol (F7); Figure S3: 1H-NMR of methylene chloride: methanol (F8). Table S1: Plackett–Burman matrix and growth response of *Anabaena variabilis* to different medium runs (dry wt. (g L-1)). The % of increase (+) and decrease (−) in dry weight of each run compared to control (run 21) was also given; Table S2: Chemical constituents of the TLC fraction F7 of Anabaena varibilis.
