# Supplementary material for: Growth Optimization and Secondary Metabolites Evaluation of Anabaena variabilis for Acetylcholinesterase Inhibition Activity
Source: Plants (Basel). 2022 Mar 10;11(6):735. doi: 10.3390/plants11060735 (PMC8948897; doi:10.3390/plants11060735)
Supplement: Supplementary file 1 [file plants-11-00735-s001.zip › plants-1597888-supplementary.pdf]

Supporting information
Supplementary methods and results

Thin layer chromatography (TLC)

TLC separation of each effluent was carried out by spotting liquid samples of each effluent separately on silica gel Merck GF 254 per coated plates 20 × 20 cm on aluminum sheets [1]. TLC plates were vertically immersed separately in glass jars containing eluents from hexane/ethyl acetate with different polarities corresponding to each effluent. Effluents were combined into fractions according to their TLC patterns and the final fractions (F1 to F10) were tested for their inhibition against AChE. The most promising fractions were further analyzed.

Nuclear magnetic resonance (NMR)

The ^{1}H NMR spectra were recorder on a varian Mercyry VX-300 NMR spectrometer. ^{1}H NMR spectra were run at 300 MHz in deuterated chloroform (CDCl3). Chemical shifts are quoted in δ relative to that of the solvents.

GC/MS analysis

Aglient 6890 gas chromatograph equipped with an Aglient mass spectrometric detector, with a direct capillary interface and fused silica capillary column PAS-5 ms (30 m x 0.32mm x 0.25 μm film thickness) was used for the analysis of the fractions. Samples were injected in a condition at which Helium was used as carrier gas at approximately 1.0 ml/min., pulsed split less mode. The solvent delay was 3 min. and the injection size was 1.0 μl. The mass spectrophotometric detector was operated in electron impact ionization mode with an ionizing energy of 70 e.v. scanning from m/z 50 to 500. The ion source temperature was 230°C. The electron multiplier voltage (EM voltage) was maintained at 1250 V above auto tune. The instrument was manually tuned using perfluorotributyl amine (PFTBA). The GC temperature program was started at 60°C (2 min) then elevated to 300°C in a rate of 5°C/min. The injector temperature was set at 280°C. Data of Aglient 6890 gas chromatograph was analyzed by MassHunter . Wiley and Wiley Nist mass spectral data base was used in the identification of the separated peaks.

Library Search Report

Data Path : C:\msdchem\1\data\dena aly\
Data File : 00101001.D
Acq On : 3 Dec 2015 22:43
Operator :
Sample : sample 7 dena aly
Misc :
ALS Vial : 1 Sample Multiplier: 1

Search Libraries: C:\Database\W9N11.L Minimum Quality: 50
C:\Database\W9N11.L Minimum Quality: 0

Unknown Spectrum: Apex
Integration Events: ChemStation Integrator - autoint1.e

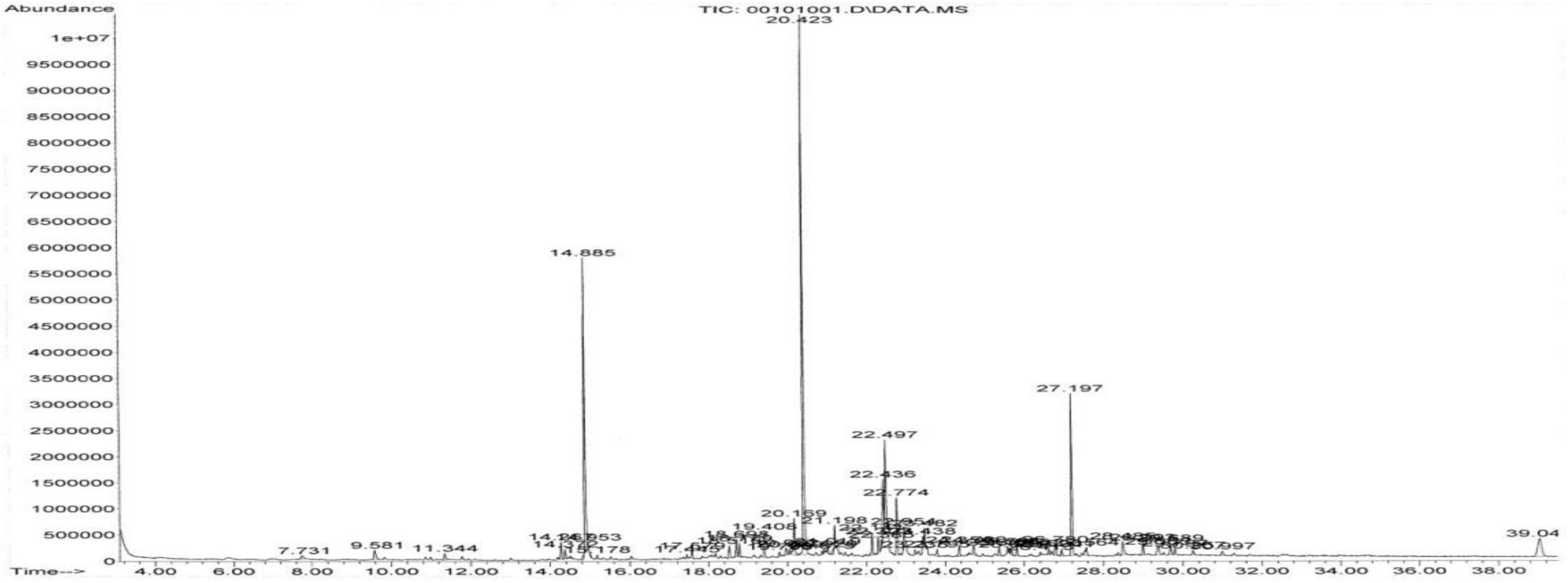

FIGER PRINT.M Fri Dec 04 00:11:42 2015 Page: 1

Figure S1: GC chromatogram of fraction F7

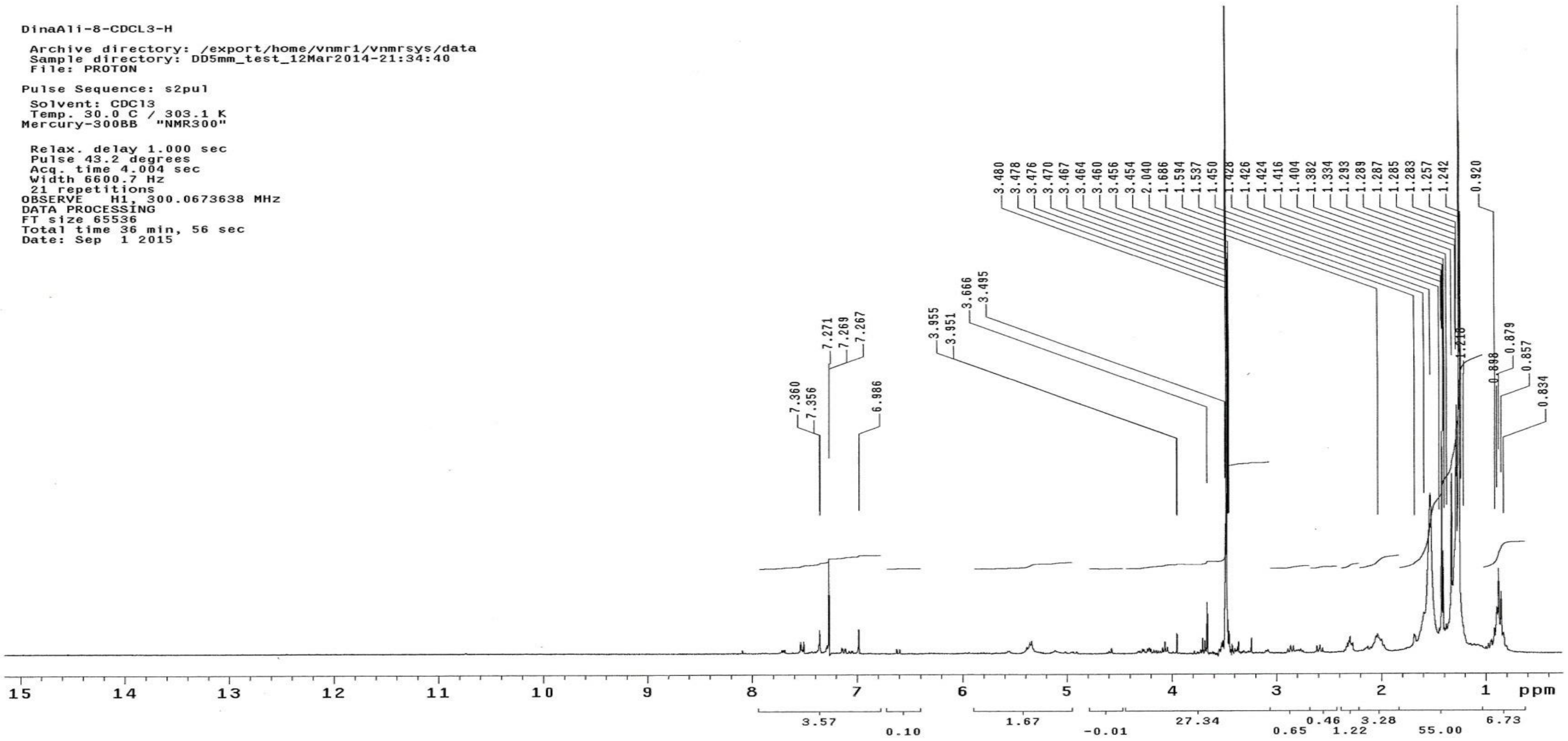

Figure S2: 1H-NMR of methylene chloride : methanol (F7)

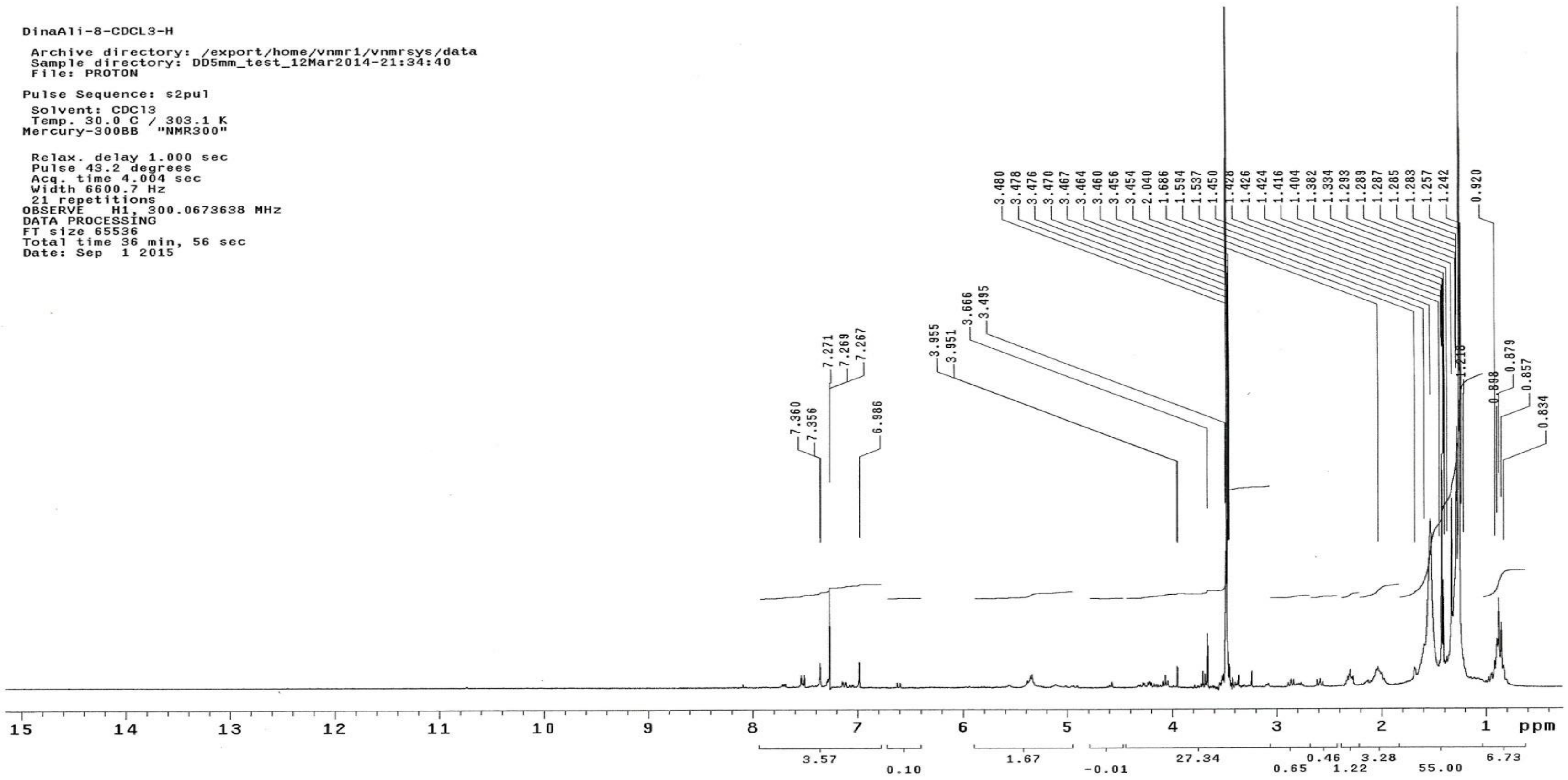

Figure S3: 1H-NMR of methylene chloride : methanol (F8).

Table S1: Plackett-Burman matrix and growth response of *Anabaena variabilis* to different medium runs (dry wt. (g L^{-1})). The % of increase (+) and decrease (-) in dry weight of each run compared to control (run 21) was also given.

Runs	X1	X2	X3	X4	X5	X6	X7	X8	X9	X10	X11	X12	X13	X14 (Dummy)	Dry wt. (g L^{-1})	% increase or decrease
1	1	-1	1	1	-1	-1	-1	-1	1	-1	1	-1	1	1	1.219	19.6 (+)
2	1	1	-1	1	1	-1	-1	-1	-1	1	-1	1	-1	1	1.206	18.4 (+)
3	-1	1	1	-1	1	1	-1	-1	-1	-1	1	-1	1	-1	1.267	24.3 (+)
4	-1	-1	1	1	-1	1	1	-1	-1	-1	-1	1	-1	1	1.483	45.5 (+)
5	1	-1	-1	1	1	-1	1	1	-1	-1	-1	-1	1	-1	1.481	45.3 (+)
6	1	1	-1	-1	1	1	-1	1	1	-1	-1	-1	-1	1	1.247	22.4 (+)
7	1	1	1	-1	-1	1	1	-1	1	1	-1	-1	-1	-1	1.192	16.9 (+)
8	1	1	1	1	-1	-1	1	1	-1	1	1	-1	-1	-1	1.606	57.6 (+)
9	-1	1	1	1	1	-1	-1	1	1	-1	1	1	-1	-1	3.353	229.1 (+)
10	1	-1	1	1	1	1	-1	-1	1	1	-1	1	1	-1	1.008	-1.07 (-)
11	-1	1	-1	1	1	1	1	-1	-1	1	1	-1	1	1	0.878	-13.84 (-)
12	1	-1	1	-1	1	1	1	1	-1	-1	1	1	-1	1	3.622	255.4 (+)
13	-1	1	-1	1	-1	1	1	1	1	-1	-1	1	1	-1	4.028	295.3 (+)
14	-1	-1	1	-1	1	-1	1	1	1	1	-1	-1	1	1	1.733	70 (+)
15	-1	-1	-1	1	-1	1	-1	1	1	1	1	-1	-1	1	1.569	53.9 (+)
16	-1	-1	-1	-1	1	-1	1	-1	1	1	1	1	-1	-1	2.444	139.84 (+)
17	1	-1	-1	-1	-1	1	-1	1	-1	1	1	1	1	-1	1.372	34.6 (+)
18	1	1	-1	-1	-1	-1	1	-1	1	-1	1	1	1	1	0.950	-6.8 (-)
19	-1	1	1	-1	-1	-1	-1	1	-1	1	-1	1	1	1	1.203	18.1 (+)
20	-1	-1	-1	-1	-1	-1	-1	-1	-1	-1	-1	-1	-1	-1	1.250	22.7 (+)
21	0	0	0	0	0	0	0	0	0	0	0	0	0	0	1.019	0

Table S2: Chemical constituents of the TLC fraction F7 of *Anabaena varibilis*.

Peak No.[a]	Retention time (min) [b]	Identification (IUPAC nomenclature)	Compound No. [c]	Formula	$[M]^{+}$ [d]	Area %
1	7.733	Undecane	(1)	$C_{11}H_{24}$	156	0.37
2	9.579	Dodecane	(2)	$C_{12}H_{26}$	170	0.95
3	11.343	Tridecane	(3)	$C_{13}H_{28}$	184	0.52
8	15.180	Eicosane	(10)	$C_{20}H_{42}$	282	0.26
10	17.579	2-(octadecycloxy) ethanol	(37)	$C_{20}H_{42}O_2$	314	0.53
12	18.697	1-Octadecene	(17)	$C_{18}H_{36}$	252	1.02
13	18.778	Octadecane	(8)	$C_{18}H_{38}$	254	0.73
14	19.407	6,10,14-trimethyl-2-pentadecanone	(26)	$C_{18}H_{36}O$	268	1.53
16	19.960	Benzoic acid, 2-hydroxy-,phenylmethyl ester	(40)	$C_{14}H_{12}O_3$	228	0.54
17	20.047	Nonadecane	(9)	$C_{19}H_{40}$	268	0.28
18	20.170	9-hexadecenoic, methyl ester	(27)	$C_{17}H_{32}O_2$	268	2.47
19	20.420	Hexadecanoic, methyl ester	(28)	$C_{17}H_{34}O_2$	270	31.26
20	20.874	Heneicosane	(11)	$C_{21}H_{44}$	296	0.17
23	21.200	3-Eicosene	(18)	$C_{20}H_{40}$	280	0.28
24	22.143	5,7-dihydroxy-2-phenyl-4H-chromen-4-one	(41)	$C_{15}H_{10}O_4$	254	1.38
25	22.301	15-Heptadecenal	(25)	$C_{17}H_{32}O$	252	0.94
26	22.388	1,9-hexadecadiene	(20)	$C_{16}H_{30}$	222	0.36
27	22.435	9,12-octadecadienoic acid (z,z)-methyl ester	(32)	$C_{19}H_{34}O_2$	294	2.55
28	22.499	9-octadecenoic acid(z), methyl ester	(29)	$C_{19}H_{36}O_2$	296	4.81
29	22.772	Octadecanoic acid, methyl ester	(30)	$C_{19}H_{34}O_2$	298	3.14
32	23.436	7-isopropyl-4-methyl-6,7,8,8a-tetrahydroazulen-1-(5H)-one	(44)	$C_{15}H_{22}O$	218	0.31
33	23.483	1-docosene	(19)	$C_{22}H_{44}$	308	0.74
34	23.797	Isophytol, acetate	(38)	$C_{22}H_{42}O_2$	338	0.47

35	24.356	2,5-dihydroxybenzoic acid	(39)	$C_8H_6O_4$	166	0.69
39	25.683	Hexanedioic acid, bis(2-ethylhexyl) ester	(34)	$C_{22}H_{42}O_2$	370	0.30
41	26.411	Hexanoic acid, 2-ethyl-hexadecyl ester	(33)	$C_{24}H_{48}O_2$	368	0.21
44	26.941	2H,8H-Benzo[1,2-b:5,4-b']dipyran-10-propanol, 5-methoxy-2,2,8,8-tetramethyl	(45)	$C_{20}H_{26}O_4$	330	0.37
46	27.564	Tricosane	(13)	$C_{23}H_{48}$	324	0.72
54	39.040	4-phenyl-2-(pyridin-3-yl)quinazoline	(46)	$C_{19}H_{13}N_3$	283	3.45

[a] number in GC chromatogram
[b] compounds are arranged according to their ascending retention time
[c] number shown in Figure S1
[d] molecular weight
